# Targeted cholangioscopy-guided biopsies through the mesh of biliary metal stents – a challenging diagnosis of perihilar cholangiocarcinoma

**DOI:** 10.1055/a-2738-7015

**Published:** 2025-11-19

**Authors:** Ivo Mendes, Francisco Vara-Luiz, Carolina Palma, Filipe Nogueira, Júlio Veloso, Jorge Fonseca, Gonçalo Nunes

**Affiliations:** 1 70816 Gastroenterology Department, Hospital Garcia de Orta, Almada, Portugal; 2 Aging Lab, Egas Moniz Center for Interdisciplinary Research (CiiEM), Egas Moniz School of Health and Science, Almada, Portugal; 3 70816 Pathology Department, Hospital Garcia de Orta, Almada, Portugal

A 59-year-old man presented with painless jaundice. Laboratory data revealed an alanine aminotransferase of 95 UI/L, an aspartate aminotransferase of 54 UI/L, an alkaline phosphatase of 550 UI/L, a total bilirubin of 6 mg/dL and a direct bilirubin of 4.6 mg/dL. Computed tomography (CT) showed a 4 cm unresectable perihilar infiltrative lesion with portal vein invasion and cavernous transformation. Ultrasound-guided biopsy and percutaneous transhepatic cholangiography were performed, allowing successful palliative biliary drainage with non-covered metal stents (one on the left and two on the right hepatic ducts). Nevertheless, histopathology of the sample showed no intersected neoplastic tissue.

Given the strong suspicion of malignancy and the need for histopathological tumor confirmation to guide systemic treatment, endoscopic retrograde cholangiopancreatography with cholangioscopy-guided biopsies was proposed ( Video 1 ). Following conventional biliary cannulation with a 0.035’’ guidewire, three metallic stents crossing the hilum were observed in the cholangiography ( Fig. 1 ). After biliary sphincterotomy, a 3.2 mm cholangioscope (EyeMAX, Microtech) was introduced. The common bile duct and both hepatic ducts were carefully explored advancing the cholangioscope through the stents into the intrahepatic branches ( Fig. 2 ). Detailed inspection identified areas of pseudopolypoid appearance covering the stent body in the hilar region with marked friability and aberrant vascularization suggesting invasive neoplasia ( Fig. 3 ). Cholangioscopy-guided biopsies were laboriously performed through the stent mesh using a 1mm biopsy forceps (EyeMAX biopsy forceps, Microtech). No stent displacement or damage occurred. A 7-french nasobiliary tube was placed due to persistent haemobilia after biopsies, allowing saline flushing for 24–48 h ( Fig. 4 ). The patient was further discharged asymptomatic and antibiotic prophylaxis maintained for 5 days. Histopathology confirmed a moderately differentiated cholangiocarcinoma ( Fig. 5 ), and palliative chemotherapy could be started.

Targeted cholangioscopy-guided biopsies through the mesh of biliary metal stents.Video 1

**Fig. 1:**
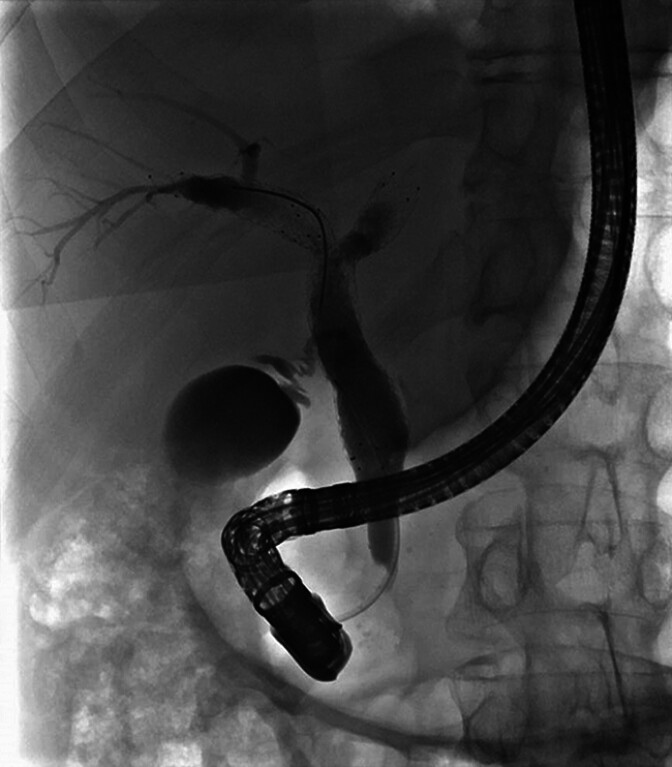
Cholangiography revealing no biliary duct dilation with three metallic stents *in situ* crossing the hilum.

**Fig. 2:**
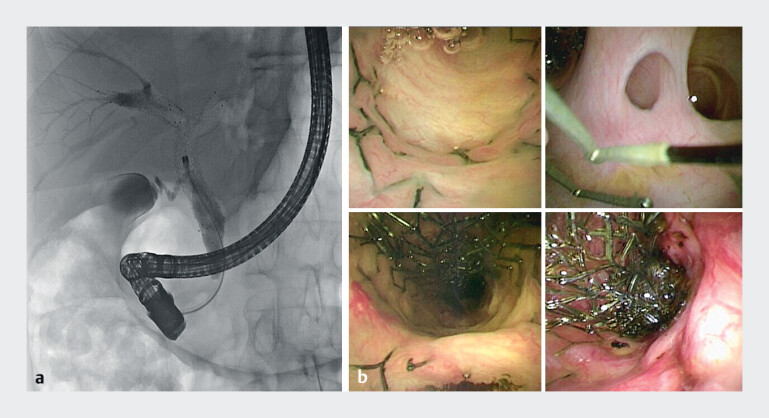
The common bile duct and the right and left hepatic ducts were carefully explored advancing the cholangioscope through the stents into the intrahepatic ducts: **a** fluoroscopic vision and **b** cholangioscopy vision.

**Fig. 3:**
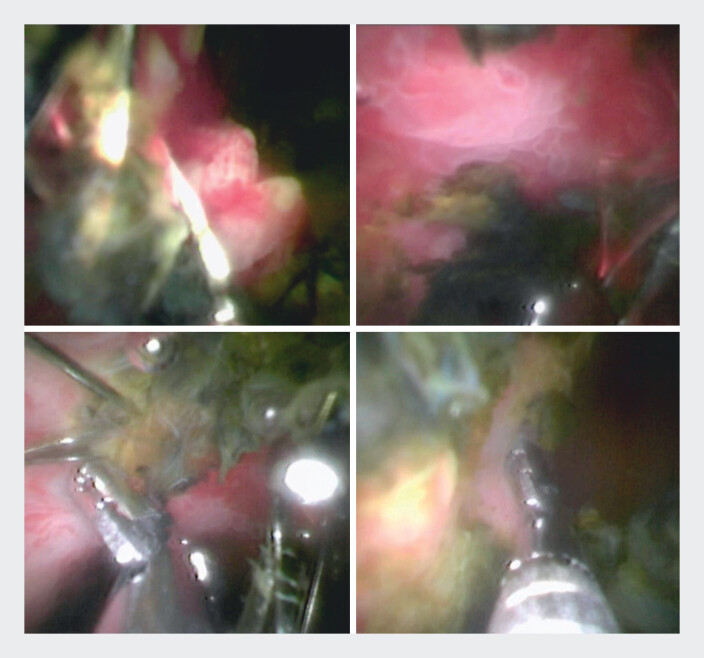
Areas of pseudopolypoid appearance in the hilar region with marked friability and aberrant vascularization were identified and cholangioscopy-guided biopsies were laboriously performed through the mesh of the metal stents.

**Fig. 4:**
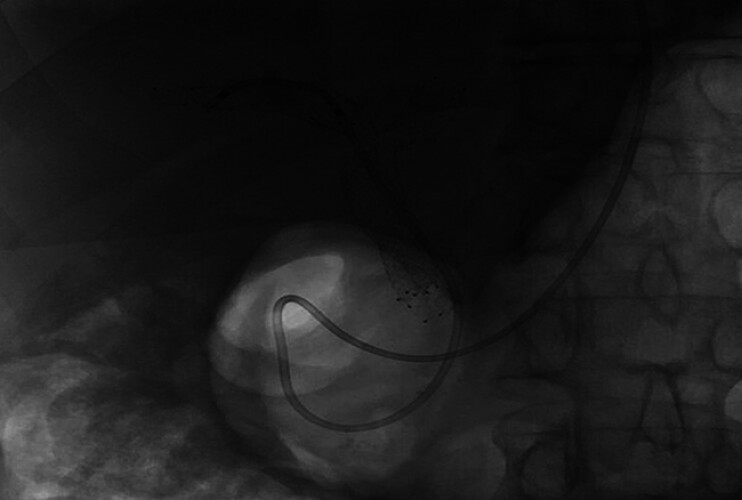
A 7-french nasobiliary tube was placed due to persistent haemobilia after biopsies, allowing flushing with saline for 24–48 h.

**Fig. 5:**
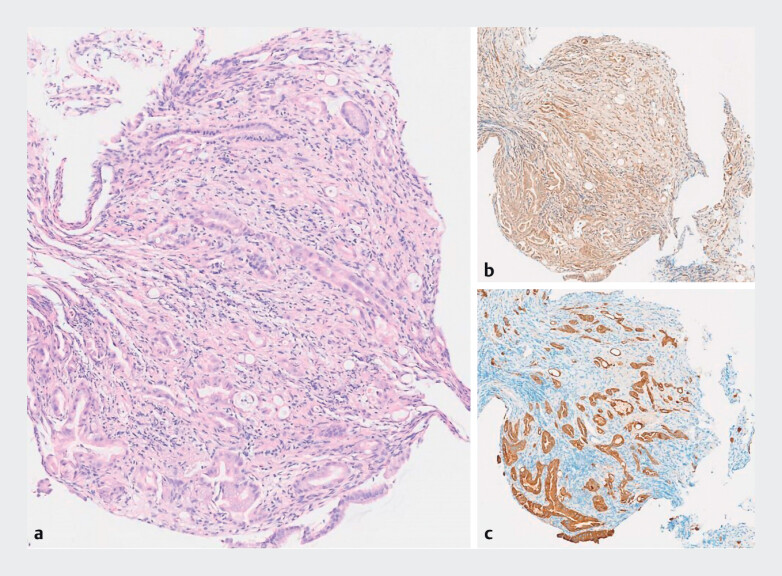
Histopathology confirmed a moderately differentiated cholangiocarcinoma: **a** hematoxylin and eosin; **b** preserved SMAD4 expression; and **c** CK19 expression).

This case highlights the value of cholangioscopy-guided biopsies in the challenging diagnosis of perihilar malignancies. Non-covered metal stents should only be used after a definite histologic confirmation of cancer, as further removal is virtually impossible. Despite the potential technical limitations of this unreported approach, targeted biopsies through the stent mesh seem effective without compromising stent integrity.

Endoscopy_UCTN_Code_TTT_1AR_2AD

## Notes

Endoscopy E-Videos https://eref.thieme.de/e-videos
*E-Videos* is an open access online section of the journal *Endoscopy* , reporting on interesting cases and new techniques in gastroenterological endoscopy. All papers include a high-quality video and are published with a Creative Commons CC-BY license. Endoscopy E-Videos qualify for HINARI discounts and waivers and eligibility is automatically checked during the submission process. We grant 100% waivers to articles whose corresponding authors are based in Group A countries and 50% waivers to those who are based in Group B countries as classified by Research4Life (see: https://www.research4life.org/access/eligibility/ ). This section has its own submission website at https://mc.manuscriptcentral.com/e-videos .

